# Adrenomedullin and truncated peptide adrenomedullin(22-52) affect chondrocyte response to apoptotis in vitro: downregulation of FAS protects chondrocyte from cell death

**DOI:** 10.1038/s41598-020-73924-1

**Published:** 2020-10-07

**Authors:** Frédéric Velard, Aurore Chatron-Colliet, Dominique Côme, Marie-Dominique Ah-Kioon, Hilène Lin, Narjes Hafsia, Martine Cohen-Solal, Hang-Korng Ea, Frédéric Lioté

**Affiliations:** 1 INSERM, UMR-S 1132 Bioscar, Centre Viggo Petersen, Hôpital Lariboisière, 2, Rue Ambroise Paré, 75010 Paris, France; 2 Université de Paris (UFR de Médecine), 75205 Paris, France; 3 Assistance Publique-Hôpitaux de Paris (AP-HP), Service de Rhumatologie, Centre Viggo Petersen, Hôpital Lariboisière, 75010 Paris, France

**Keywords:** Rheumatic diseases, Cell biology, Rheumatology

## Abstract

Chondrocyte apoptosis may have a pivotal role in the development of osteoarthritis. Interest has increased in the use of anti-apoptotic compounds to protect against osteoarthritis development. In this work, we investigated the effect of adrenomedullin (AM), a 52 amino-acid hormone peptide, and a 31 amino-acid truncated form, AM(22-52), on chondrocyte apoptosis. Bovine articular chondrocytes (BACs) were cultured under hypoxic conditions to mimic cartilage environment and then treated with Fas ligand (Fas-L) to induce apoptosis. The expression of AM and its calcitonin receptor-like receptor (CLR)/receptor activity-modifying protein (RAMP) (receptor/co-receptor) was assessed by immunostaining. We evaluated the effect of AM and AM(22-52) on Fas-L-induced chondrocyte apoptosis. FAS expression was appreciated by RT-qPCR and immunostainings. The expression of hypoxia-inducible factor 1α (HIF-1α), CLR and one co-receptor (RAMP2) was evidenced. With BACs under hypoxia, cyclic adenosine monophosphate production increased dose-dependently with AM stimulation. AM significantly decreased caspase-3 activity (mean 35% decrease; *p* = 0.03) as a marker of Fas-L-induced apoptosis. Articular chondrocytes treated with AM showed significantly reduced cell death, along with downregulated Fas expression and production, as compared with AM(22-52). AM decreased articular chondrocyte apoptosis by downregulating a Fas receptor. These findings may pave the way for novel therapeutic approaches in osteoarthritis.

## Introduction

Cartilage is a tissue that is synthesized and maintained by a unique cell type, namely the chondrocyte. Osteoarthritis (OA) is a debilitating joint disease characterized by a progressive destruction of the articular cartilage after, at least in part, chondrocyte apoptosis, which has been postulated to have a pivotal role in the development of OA^1^.

Adrenomedullin (AM) is a 52 amino-acid peptide that belongs to the calcitonin family^2^. First discovered in human pheochromocytoma^3^, AM is expressed in many tissues (cardiovascular system, brain, kidneys, lungs, and also bone and joint)^4–6^. Murine and human articular chondrocytes also express AM receptors^4,5^.

AM is known for its angiogenic effect but also has anti-apoptotic features^7,8^ and modulates cell proliferation and cell migration^9,10^. AM mediates its functions by interacting with a heterodimeric membrane receptor (calcitonin receptor-like receptor [CLR] coupled to receptor activity-modifying protein 2 [RAMP2] or 3), which mainly triggers the G-protein-coupled adenylate cyclase/protein kinase A pathway^11,12^. Binding to the receptor and signal transduction are ensured by two important structures: (1) an intra-molecular loop between cysteine residues 16 and 21, and (2) an amidated C-terminal tyrosine residue. The 31 amino-acid truncated form, AM(22-52), does not have the six-amino acid ring and acts on the AM receptor as an antagonist on fibroblast-like synoviocytes^8^ or as an agonist on immortalized osteoblastic cells^13^. Of note, different effects may be attributed to AM itself depending on the models. Both AM and AM(27-52) have been shown to increase bone mass and possibly cartilage thickness in normal rats^4,14^, whereas AM inducible knock out or pharmacological inhibition in adult mice has been evidenced to increase bone mass^15^.

AM and AM(22-52) also have immunoregulatory properties in collagen-induced arthritis (CIA)^16,17^. Using the murine CIA model, we demonstrated an anti-apoptotic effect of AM and AM(22-52) on articular chondrocytes, which helped decrease the severity of the experimental arthritis^17^. We thus hypothesized that both peptides might affect chondrocyte death in isolated OA chondrocytes.

Here, we investigated whether AM or AM(22-52) could regulate apoptosis in vitro in bovine articular chondrocytes (BACs) under specific hypoxic or normoxic conditions.

## Results

### Expression of functional AM receptor complex CLR/RAMP2 is upregulated under hypoxia in BACs in vitro

**Figure 1:**
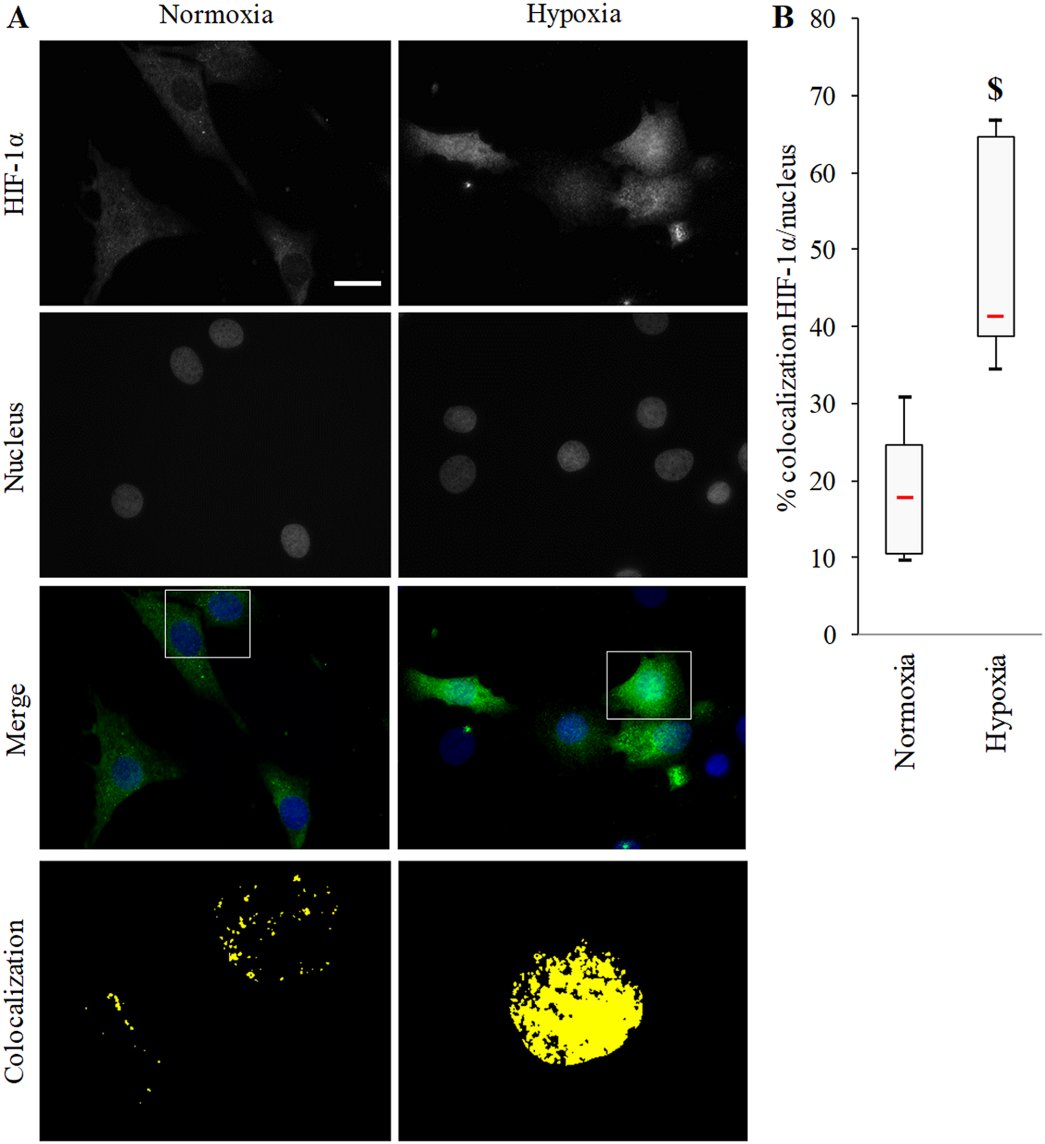
Hypoxia-inducible factor 1α (HIF-1α) localization in bovine articular chondrocytes (BACs). (**A**) Representative immunofluorescence micrographs showing hypoxia-inducible factor 1α (HIF-1α; AlexaFluor488) localization in bovine articular chondrocytes (BACs) under atmospheric O_2_ content (normoxia) or hypoxia (3% O_2_). Nuclei were counterstained with DAPI. White scale bar = 20 μm. (Objective × 63 Zeiss AxioObserver Z1 AxioCam MR3). Last line shows HIF-1α/nuclei colocalization enlargements (from white frames on merged images). (**B**) Quantification of colocalized pixels on green and blue (DAPI) channels. Experiments involved at least 7 animals. With five randomly chosen fields, measurements involved 201 and 278 cells for hypoxia and normoxia conditions, respectively. $ *p* < 0.05 compared with normoxia. Red bar represents median value. Black bars represent first and ninth deciles and limits of white rectangle represent first and third quartiles.

To mimic physiological conditions in vitro, we first validated 3% O_2_ culture model as a hypoxic culture model for BACs. We followed HIF-1α translocation through the nuclei of BACs as a marker of cellular response to hypoxia. In normoxia-cultured cells, HIF-1α expression was detected in cytoplasm, which was reinforced under hypoxia (Fig. 1A). Moreover, hypoxia promoted nuclear localization, reaching a significant threefold mean increase (*p* = 0.004) in HIF-1α/nucleus colocalization (Fig. 1B).

**Figure 2:**
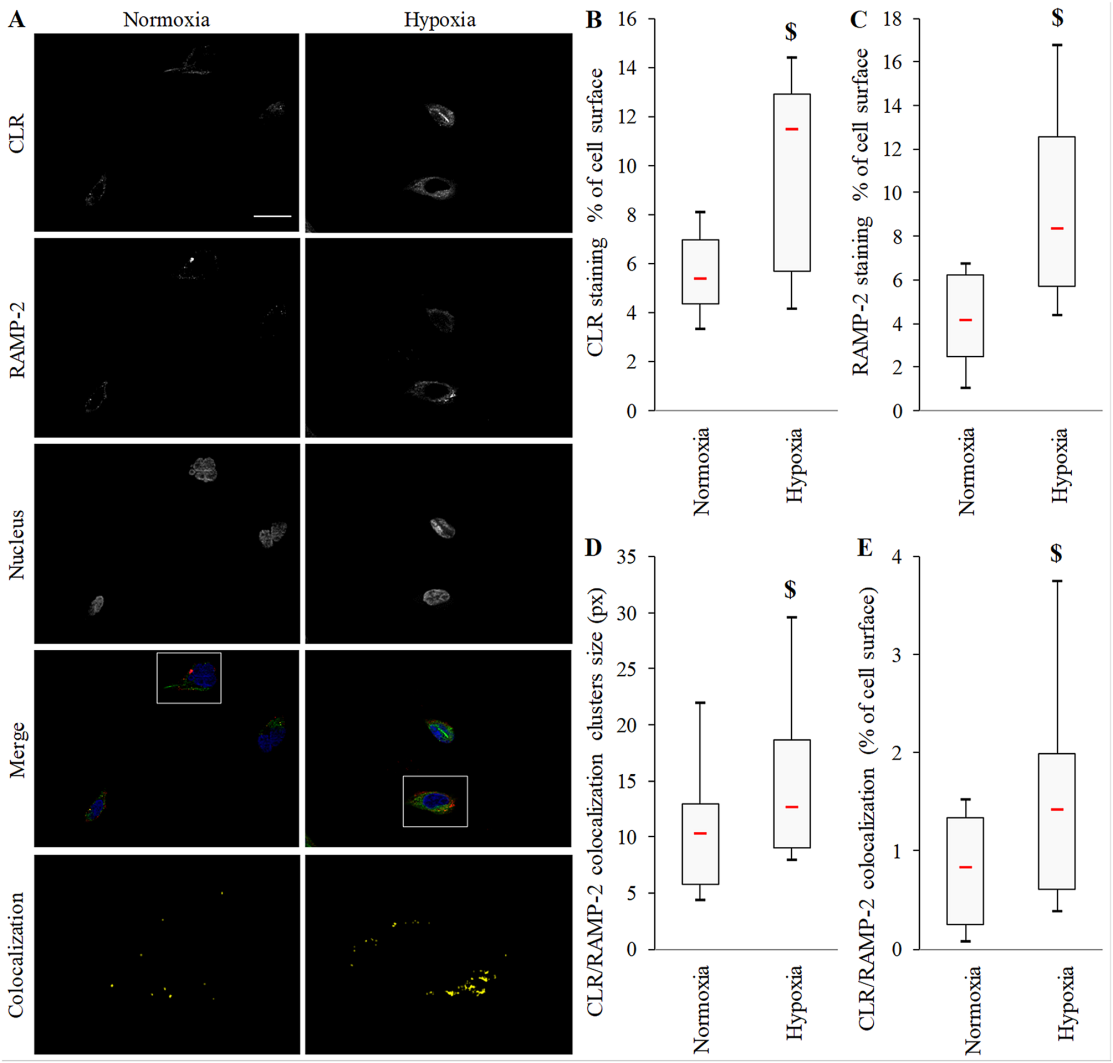
Immunolocalization of calcitonin receptor-like receptor (CLR) and receptor activity-modifying protein 2 (RAMP2) in BACs under normoxia or hypoxia. (**A**) Representative immunofluorescence micrographs of localization of calcitonin receptor-like receptor (CLR; AlexaFluor488, green) and receptor activity-modifying protein 2 (RAMP2; PE, red) in BACs under atmospheric O_2_ content (normoxia) or hypoxia (3% O_2_). Nuclei were counterstained with DAPI (blue). White scale bar = 20 μm. (objective × 63 ZeissAxio Observer Z1 AxioCam MR3). Last line shows CLR/RAMP2 colocalization enlargements (from white frames on merged images). Quantification of CLR (**B**) and RAMP2 (**C**) staining, (**D**) CLR/RAMP2 colocalized pixel cluster size on green and red channels and (**E**) total CLR/RAMP2 colocalized pixels on green and red channels. Experiments involved 8 animals. With five randomly chosen fields, measurements involved 140 and 102 cells for hypoxia and normoxia conditions, respectively. $ *p* < 0.05 compared with normoxia. Red bar represents median value. Black bars represent first and ninth deciles and limits of white rectangle represent first and third quartiles.

We then investigated the expression and activity of CLR/RAMP2 AM receptor on BACs. Immunofluorescent staining revealed a more sustained signal for both CLR and RAMP2 under hypoxia versus normoxia (mean increase of 1.73- and 2.43-fold, respectively, *p* = 0.001 for both) (Fig. 2A–C). In addition, hypoxic culture increased the colocalization cluster size (mean 51% increase, *p* = 0.027) and widespread distribution at the cell surface (mean 2.32-fold increase, *p* < 0.001) (Fig. 2D,E).

**Figure 3:**
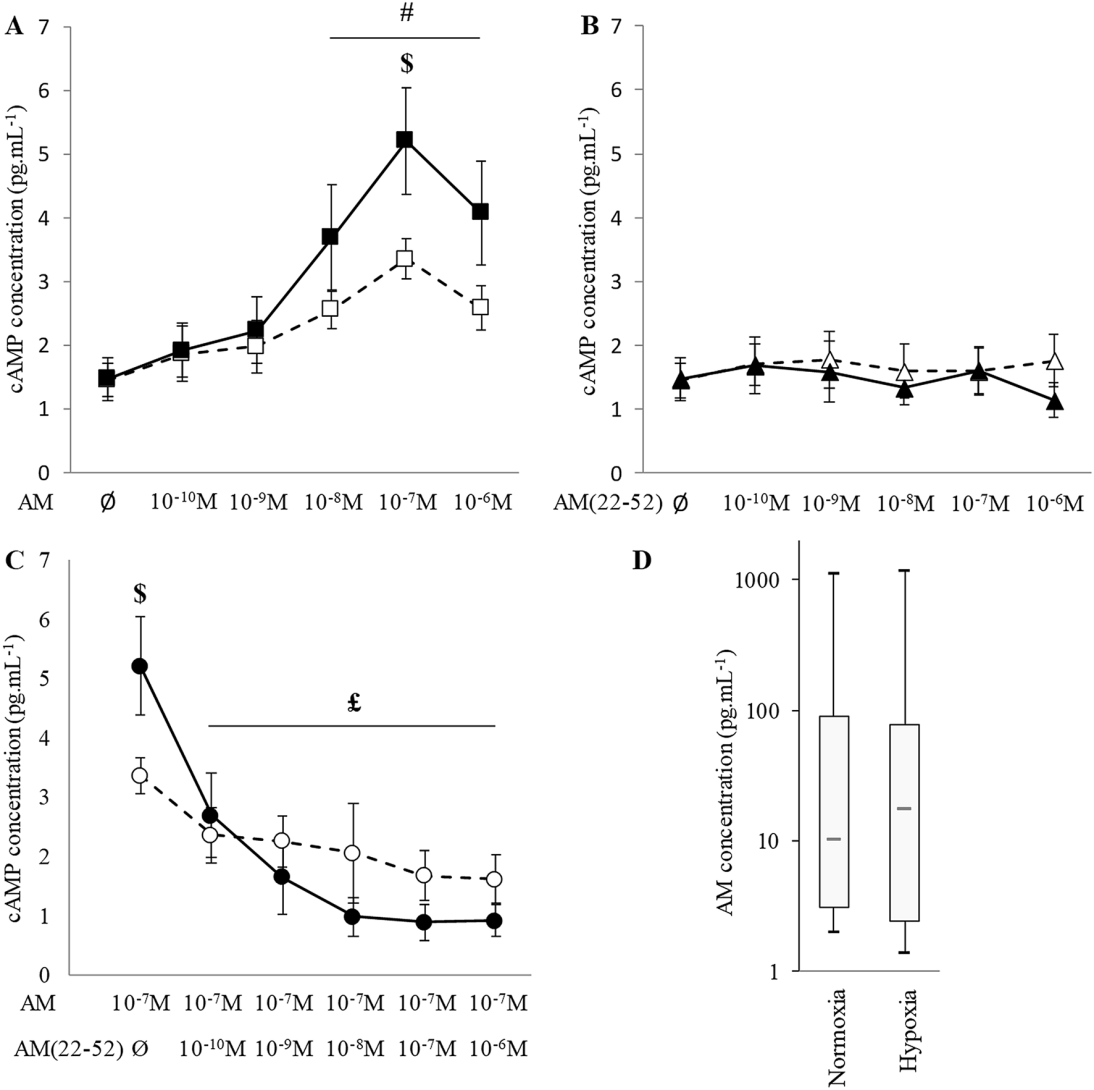
AM receptor is functional in bovine articular chondrocytes. (**A**,**B**) AM receptor function assessed by cAMP quantification in BACs under atmospheric O_2_ content (normoxia, white symbols) and 3% O_2_ hypoxia (black symbols) with increasing concentrations of AM and AM(22-52), respectively. (**C**) cAMP quantification in 10^−7^ M AM-stimulated BACs exposed to increasing concentrations of AM(22-52) under normoxia (white circles) and hypoxia (black circles). (**A**–**C**) data are mean ± SD. (**D**) AM quantification in normoxia and hypoxia-cultured BAC culture supernatant. $ *p* < 0.05 compared with normoxia. # *p* < 0.05 compared with basal condition. £ *p* < 0.05 compared with 10^−7^ M AM. Red bar represents median value. Black bars represent first and ninth deciles and limits of white rectangle represent first and third quartiles.

In both normoxic and hypoxic conditions, AM could induce cyclic adenosine monophosphate (cAMP) production in BACs via the CLR/RAMP2 receptor (Fig. 3A). Moreover, we found a dose-dependent effect of AM on cAMP production between 10^−8^ and 10^−6^ M under both normoxia (*p* = 0.016) and hypoxia (*p* = 0.031), with maximal effect at 10^−7^ M (mean 2.31- and 3.54-fold increase, respectively). At this concentration, cAMP production was higher under hypoxia than normoxia (mean 55% increase, *p* = 0.050). Conversely, AM(22-52) did not induce cAMP production at any concentration tested (*p* > 0.05) (Fig. 3B). Moreover, when added before AM 10^−7^ M stimulation, AM(22-52) blocked cAMP production (Fig. 3C). Such an effect was visible from 10^−10^ M AM(22-52) concentration, with a mean 48% reduction in cAMP content (*p* = 0.031), and culminated at 10^−7^ M, with a mean 83% reduction. Finally, we assessed the production of AM by BACs and found no significant difference between normoxia and hypoxia, with median concentrations of 10 and 20 pg mL^−1^, respectively (Fig. 3D), corresponding to approximately 10^−11^ M. Therefore, the intrinsic production of AM will not be a bias in a model in which we use 10^−7^ M of exogenous AM in BACs.

### AM regulates BAC apoptosis after Fas-L stimulation in vitro

**Figure 4:**
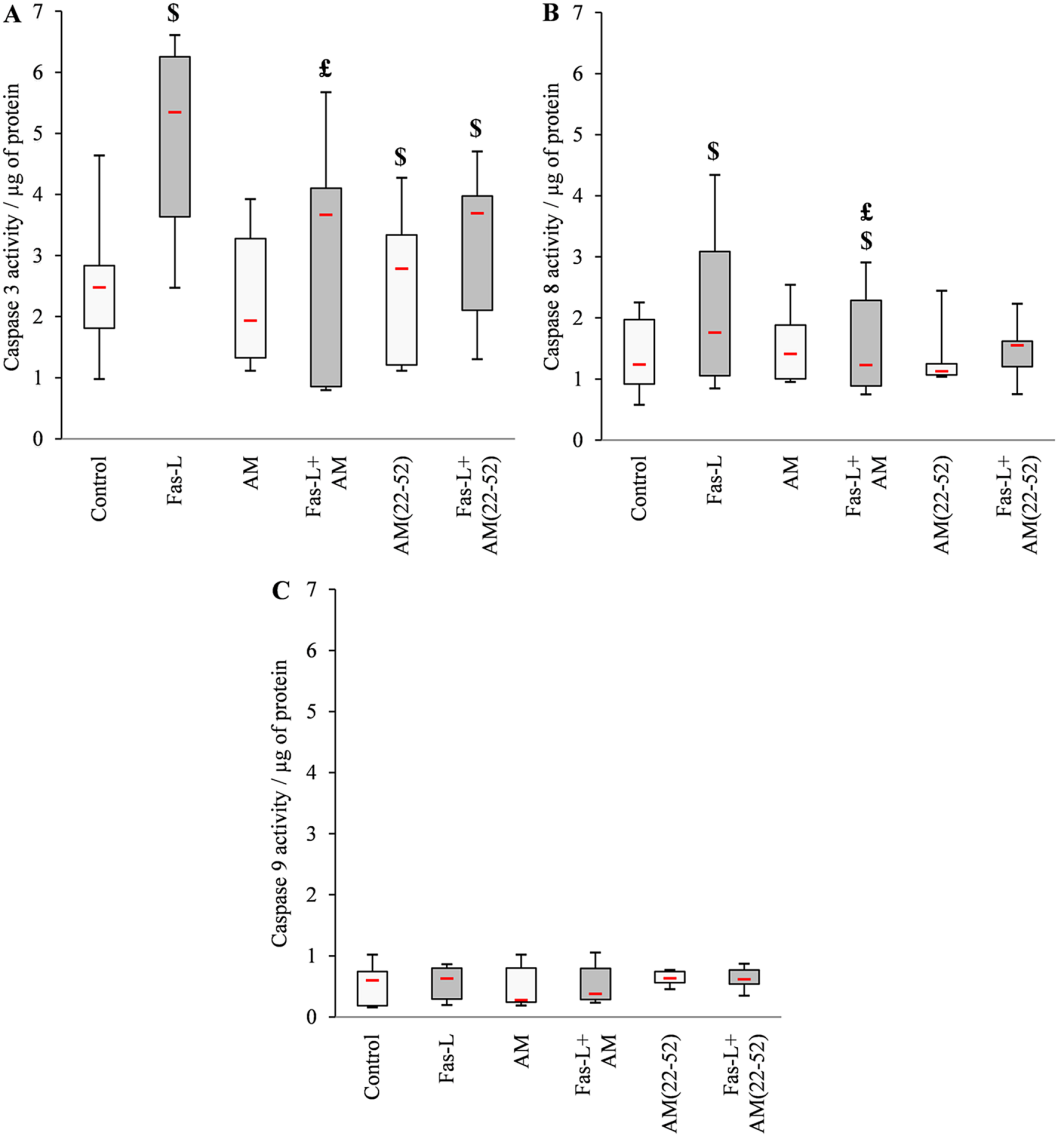
Effect of AM and AM(22-52) on early apoptosis in hypoxia-cultured BACs assessed by caspase activity measurement. (**A**) Caspase-3, (**B**) caspase-8 and (**C**) caspase-9 activities related to protein content assessed after 24 h of culture without (white) or with (gray) 20 ng mL^−1^ Fas-L. Experiments involved at least 7 animals. $ *p* < 0.05 compared with control. £ *p* < 0.05 compared with Fas-L. Red bar represents median value. Limits of white rectangle represent first and third quartiles and black bars represent first and ninth deciles.

Under hypoxia, BACs were responsive to Fas-L-induced cell death as demonstrated by increased activity of caspase-3 and -8 (mean 95% and 48% increase; *p* = 0.002 and *p* = 0.003, respectively), the major markers of apoptosis induced by death receptors (Fig. 4A,B). Of importance, caspase-9 activity (involved in the apoptosis intrinsic pathway) remained ineffective in non-stimulated or Fas-L-stimulated BACs (*p* > 0.05) (Fig. 4C). AM had no effect on caspase-3, -8 or -9 activity in the basal culture condition (*p* > 0.05), whereas AM(22-52) induced a slight but significant increase in caspase-3 activity [mean 2.48 and 2.79 for control and AM(22-52), respectively, *p* = 0.05]. Nevertheless, AM acted on Fas-L-induced apoptosis, as assessed by a significant decrease in caspase-3 activity (mean 35% decrease; *p* = 0.031), to the control level (*p* > 0.05). Such an effect of AM could be observed with caspase-8 activity, which also showed a mean 31% decrease compared to Fas-L-stimulated BACs (*p* < 0.05) but failed to return to the control level. AM(22-52) had no significant ability to decrease Fas-L induced caspase-3 activity (*p* > 0.05) and allowed for the increased caspase-3 activity versus the control condition (mean 27% increase; *p* = 0.016). AM(22-52) had triggered nor a decrease in Fas-L-induced caspase-8 activity, neither an increase as compared to control (*p* > 0.05).

**Figure 5:**
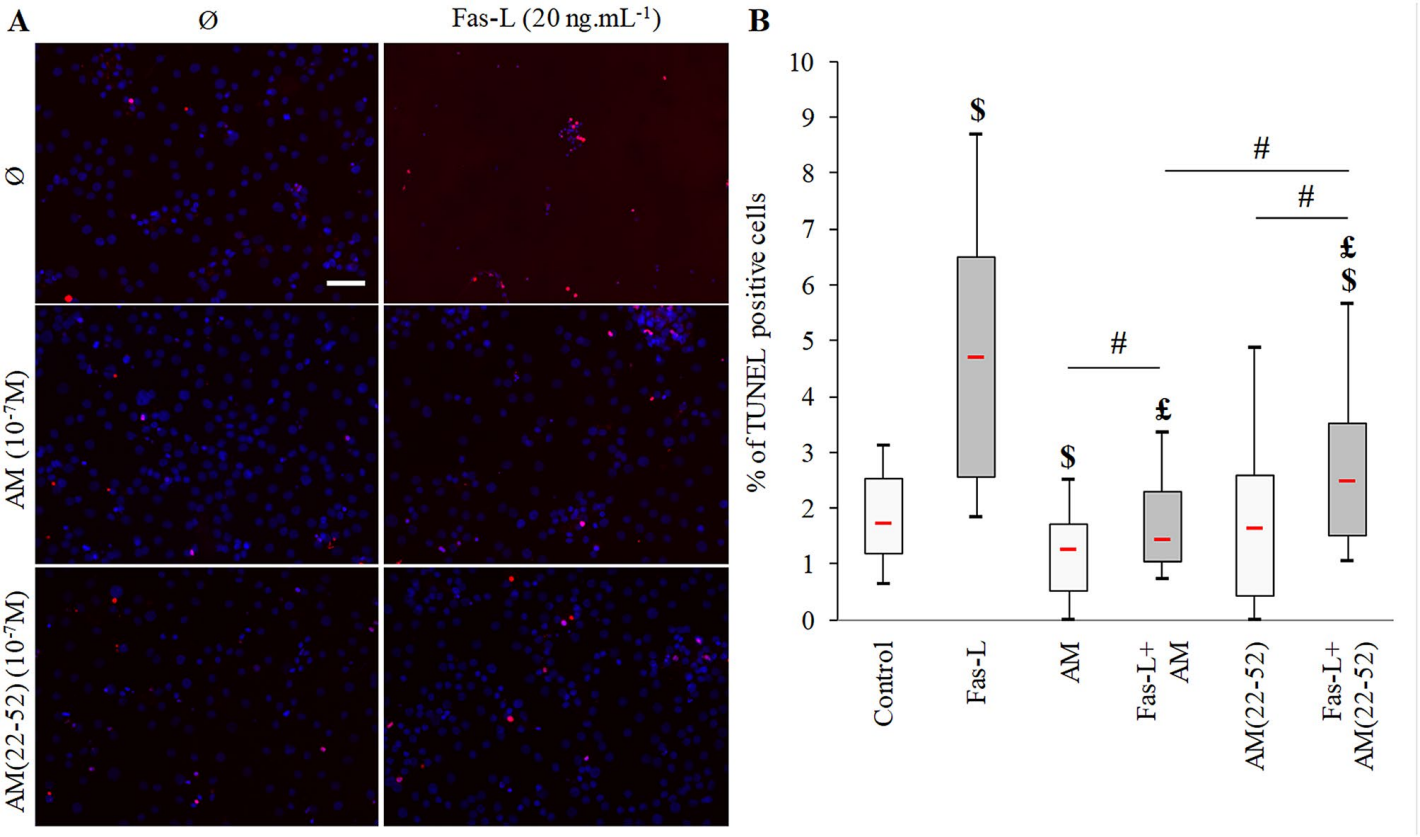
Effect of AM and AM(22-52) on late apoptosis in hypoxia-cultured BACs. (**A**) Representative photographs of fluorescent-TUNEL staining (positive cells in red) counterstained with DAPI (scale bar = 50 μm). (**B**) Percentage of TUNEL-positive cells counted on 4 randomly chosen fields after 24 h of culture without (white) or with (gray) 20 ng mL^−1^ Fas-L. Experiments involved 6 animals. A minimum of 104 cells per animal were used for analyses. $ *p* < 0.05 compared with control. £ *p* < 0.05 compared with Fas-L. # *p* < 0.05 comparing conditions. Red bar represents median value. Limits of white rectangle represent first and third quartiles and black bars represent first and ninth deciles.

**Figure 6:**
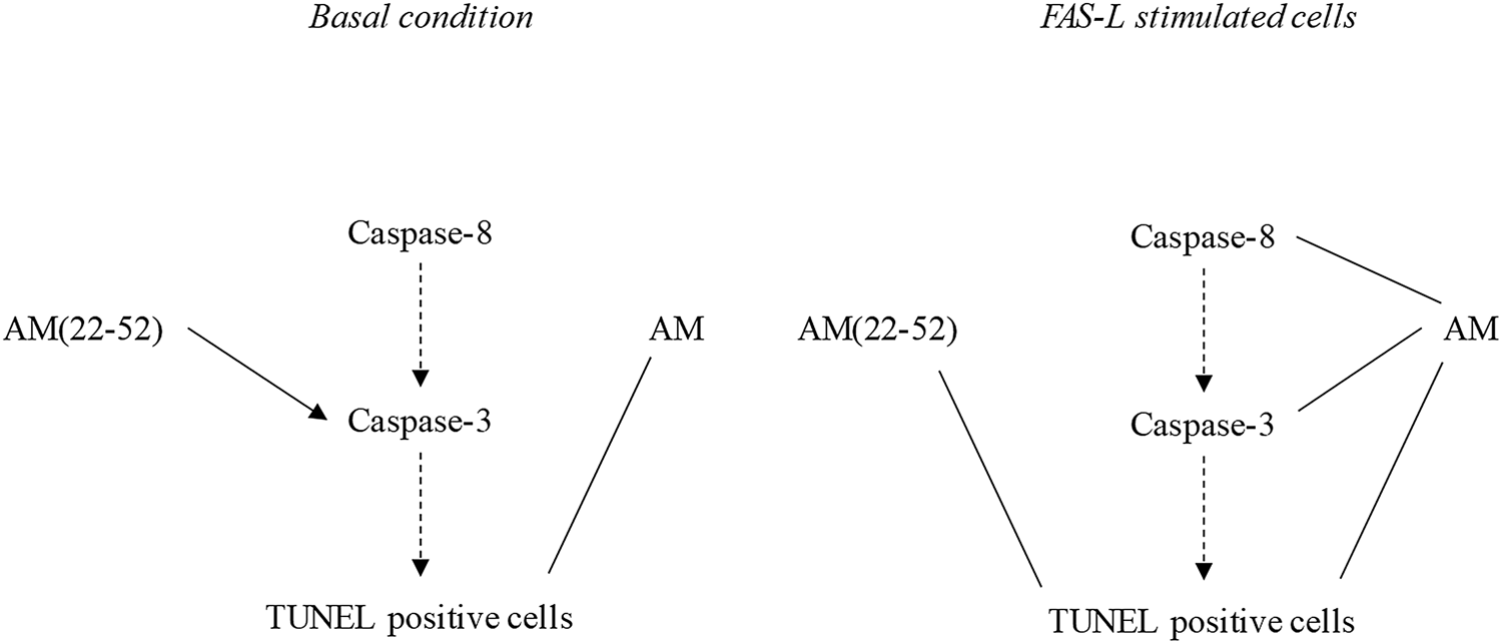
Summary of the effect of AM and AM(22-52) on BAC apoptosis in basal and Fas-L-stimulated conditions. Black arrow indicates an increase of the observed parameter, and black bar a decrease of the observed parameter.

To evaluate the effects of both AM and AM(22-52) on late stages of apoptosis, TUNEL experiments were performed to detect fragmented DNA in BACs. BACs exhibited a basal apoptosis rate (red cells, control condition), which was increased in the presence of Fas-L (Fig. 5). On quantifying the ratio of TUNEL-positive cells to total cells, we found a significant mean 2.7-fold increase in dead cell proportion versus the control condition (*p* = 0.001, Fig. 5B). Under basal culture conditions, AM induced a 36% decrease in apoptotic cells (*p* = 0.021), whereas AM(22-52) did not induce such an effect (*p* > 0.05). Of note, AM decreased the apoptotic cell number as compared with AM(22-52) (mean 40% decrease; *p* = 0.046). Of note, both AM and AM(22-52) reduced the Fas-L effect on BAC apoptosis (mean 65% and 50%, respectively; *p* < 0.001 for both). In addition, AM reduced the dead cell number to the basal level, that (22-52)AM failed to do. Moreover, the AM effect was higher than the AM(22-52) effect (mean 29% fewer apoptotic cells; *p* = 0.005). These results for AM and AM(22-52) effects on BAC apoptosis are summarized in Fig. 6.

**Figure 7:**
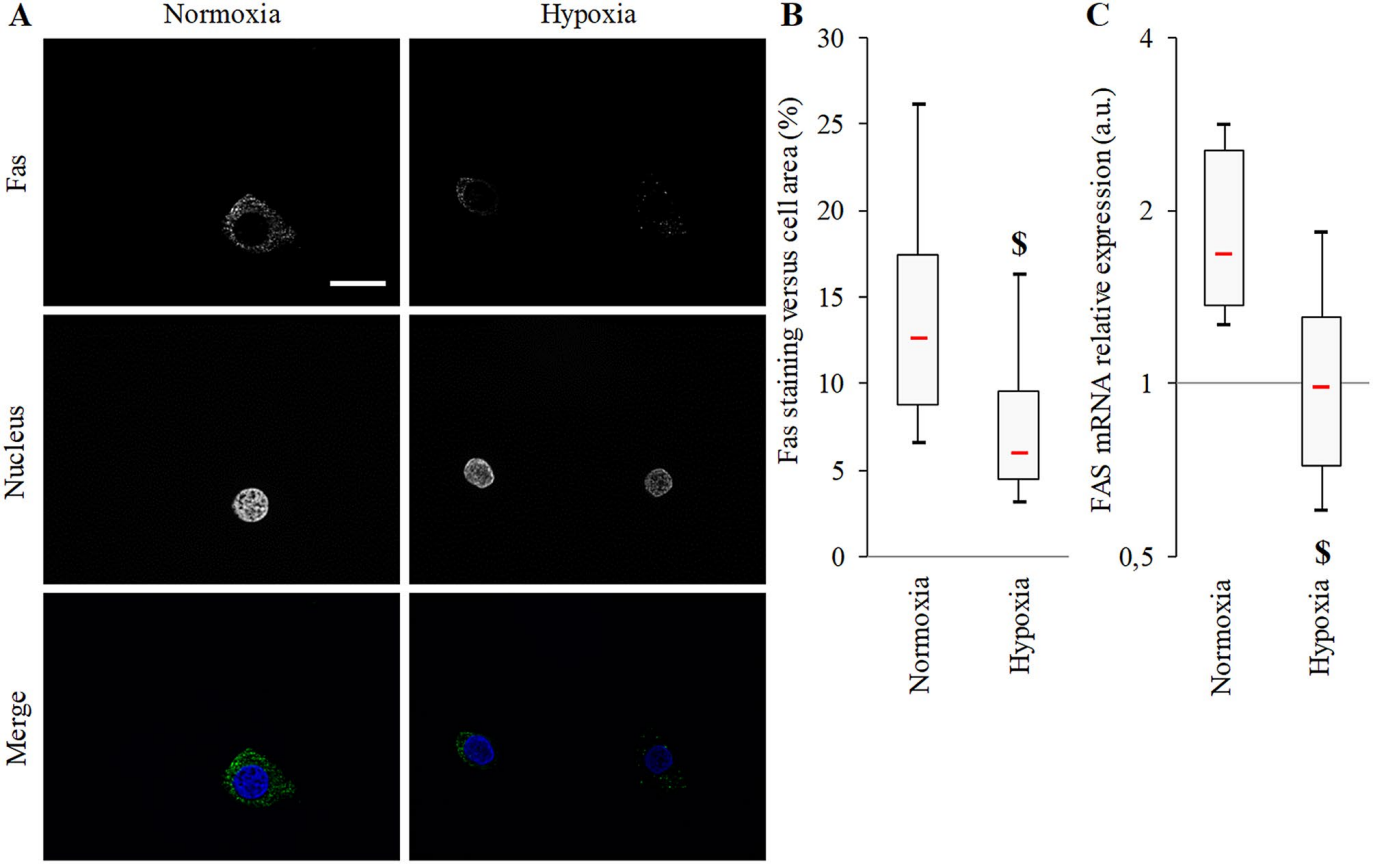
Fas localization in BACs under normoxia or hypoxia. (**A**) Fas representative immunofluorescence micrographs showing Fas (AlexaFluor488, green) localization in BACs under atmospheric O_2_ content (normoxia) or hypoxia (3% O_2_). Nuclei were counterstained with DAPI. White scale bar = 20 μm. (**B**) Fas production in BACs evaluated in immunofluorescence micrographs. (**C**) FAS mRNA expression evaluated by RT-qPCR (GAPDH was used as internal control). Experiments involved 6 animals. $ *p* < 0.05 compared with normoxia. Red bar represents median value. Limits of white rectangle represent first and third quartiles and black bars represent first and ninth deciles.

To understand how AM and AM(22-52) could enhance cellular resistance to Fas-L, we tested the Fas dysregulation hypothesis. First, the hypoxic cell culture model was tested against the classical normoxic cell culture model. Immunofluorescent staining for Fas revealed more intense cytoplasmic signal in hypoxic as compared to normoxic BACs (Fig. 7A). In line, this observation was sustained by reduced Fas distribution in the cytoplasm (mean 42% decrease; *p* = 0.031, Fig. 7B). FAS mRNA expression in BACs was decreased by a mean of 40% under hypoxia versus normoxia (*p* = 0.031, Fig. 7C), which could corroborate the decrease observed at the protein level.

**Figure 8:**
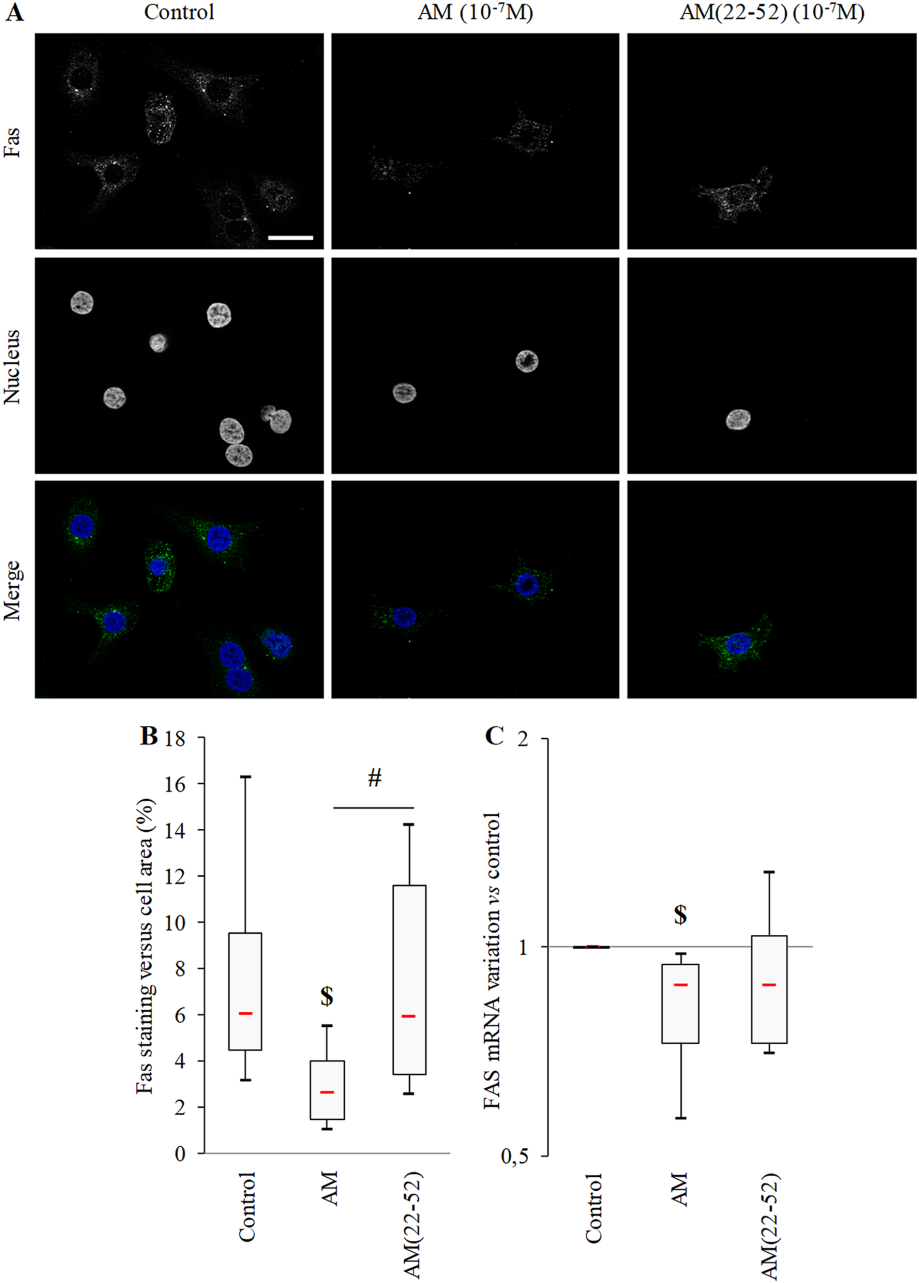
Effect of AM and AM(22-52) on Fas expression under hypoxia. (**A**) Representative immunofluorescence micrographs showing Fas (AlexaFluor488, green) localization in BACs after 24 h of culture under the indicated conditions. Nuclei were counterstained with DAPI (blue). White scale bar = 20 μm. (**B**) Fas production in BACs evaluated in immunofluorescence micrographs. (**C**) FAS mRNA expression evaluated by RT-qPCR (GAPDH was an internal control). Experiments involved at least 6 animals. $ *p* < 0.05 compared with control. # *p* < 0.05, comparing conditions. Red bar represents median value. Limits of white rectangle represent first and third quartiles and black bars represent first and ninth deciles.

Then, we used immunofluorescent staining for experiments of the AM and AM(22-52) effects on Fas expression under hypoxia (control condition) (Fig. 8A). A more sustained production of Fas was observed in AM(22-52)- versus AM-treated cells (mean 2.7-fold increase; *p* = 0.047). In addition, AM treatment decreased Fas production as compared with the control (mean 67% decrease; *p* = 0.016) (Fig. 8B). Those observations were strengthened by a mean 24% decrease in FAS mRNA expression with AM treatment (*p* = 0.031), but AM(22-52) had no effect (*p* > 0.05, Fig. 8C).

## Discussion

As compared with treating BACs with AM(22-52), AM treatment reduced caspase activities and cell death, accompanied by downregulated Fas expression and production. We bring the first piece of evidence that AM reduces articular chondrocyte apoptosis under hypoxic environment, as observed in other cellular models^1,13,18–20^.

AM was already demonstrated to exhibit immunomodulatory and anti-apoptotic properties. In the murine CIA model, AM contributes to decreased articular pro-inflammatory cytokine production and increased interleukin 10 level, a potent anti-inflammatory cytokine^17^. In addition, AM-stimulated bone-marrow–derived dendritic cells favoured regulatory T-lymphocyte induction^21^. AM was found an anti-apoptotic agent in human fibroblast-like synoviocytes and a murine osteoblastic cell line and in articular cartilage from CIA mice^8,13,17^. In many cell types, AM has also been found a critical regulator of cell survival in physiologic and pathologic environments^22–25^. To our knowledge, this paper is the first to show that AM inhibits Fas-induced apoptosis in BACs under hypoxia.

Cartilage is a non-innervated and non-vascularized tissue that is under constant hypoxia. Among classical pathways activated by low oxygen tension, hypoxia-inducible factor (HIF) is one of the most prevalent. HIF-1α is one of the two subunits of the HIF-1 complex. It is sequestered and destroyed in cell cytoplasm when O_2_ is > 5% because of its interaction with the von Hippel Lindau tumour suppressor protein (pVHL), a ubiquitin E3 ligase complex. This interaction is due to proline hydroxylation of HIF-1α by proline hydroxylase 1–3, which is reversed under 5% O_2_^26^. Additionally, pVHL is S-nitrosylated at low oxygen tension, which prevents it from interacting with HIF-1α^27^. We have validated a new culture model using 3% O_2_. The choice of this concentration was motivated by contradictory results for cell viability at 1% O_2_ and possible lack of HIF pathway expression at 5% O_2_^28,29^. Our model revealed clear nuclear translocation of HIF-1α, which suggests a functional hypoxic culture model for BACs.

In such an environment, AM production and activity could be enhanced in several cell types^23–25^, especially in endothelial cells, where CLR and AM levels were increased in response to hypoxic stress, thereby suggesting a regulatory loop to favour the proangiogenic effect of AM^7,30^. Of interest, patients with OA showed elevated levels of circulating AM, with a direct correlation with the severity of joint damage^31^. AM and AM receptor expression was previously demonstrated in chondrocytes, but to date, no information has been available on lack of oxygen modulating AM and AM receptor complex expression^5^. In the present work, we bring some evidence for AM receptor CLR/RAMP2 upregulation under hypoxia, but CLR/RAMP3 expression remained undetectable (data not shown). In addition, we demonstrated that AM production was not modified by lack of oxygen, which suggests that only CLR/RAMP2 upregulation could be considered an oxygen stress response. We also reveal that this receptor is functional. AM stimulation of BACs elevated the expression of cAMP, a second messenger that acts downstream of CLR/RAMP2 activation^4^.

Of importance in our hypoxia model, the AM(22-52) truncated peptide did not induce any effect. This peptide lacks the intracellular loop (because of a disulfide bond between cysteine residues 16–21) known to mediate the major part of the AM biological effects^3^. Moreover, AM(22-52) inhibited AM-induced cAMP production, which reveals its role as an AM antagonist. This role has been previously proposed, but results were controversial because AM(22-52) could also be an AM agonist^8,13^.

With the anti-apoptotic features of AM, this natural peptide could be used as a potential therapeutic agent against the chondrocyte apoptosis observed in OA^32,33^. In such a condition, cell death program is mainly engaged through cell death receptors such as TRAIL and Fas, which account for 20% of the cell death in OA^34,35^. Seol et al.^34^ also demonstrated that TRAIL-mediated apoptosis may be reduced under hypoxia. For the first time, we revealed a similar mechanism for Fas, which is downregulated under hypoxia, but we identified that AM but not AM(22-52) may accentuate the downregulation of the Fas receptor. This result may explain part how AM exerts its anti-apoptotic effect on Fas-L-induced BAC apoptosis.

Further work is needed to fully investigate pathways involved in AM control of chondrocyte apoptosis. However, AM may represent a potential therapeutic agent against cartilage degradation in OA.

## Methods

### Reagents

AM and its truncated peptide, AM(22-52), were purchased from GENECUST. Dulbecco's phosphate-buffered saline (DPBS), antibiotics, l-glutamine, trypsin-EDTA, Dulbecco’s modified Eagle medium (DMEM), glycerol, streptavidin-Alexa Fluor 488 and 2-(4-amidinophenyl)-1H-indole-6-carboxamidine (DAPI) were from LIFE TECHNOLOGIES. Fetal calf serum (FCS) was from PAA Laboratories Inc.; the same batch was used in all experiments. Fungizone, collagenase II, bovine serum albumin (BSA), donkey serum, Triton X-100, ethylenediaminetetraacetic acid (EDTA), phenylmethanesulfonyl fluoride (PMSF), 4-(2-hydroxyethyl)-1-piperazineethanesulfonic acid (HEPES), sulfanilamide, phosphoric acid, *N*-1-naphtylethylenediamine dihydrochloride (NED) and 3-isobutyl-1-methylxanthine (IBMX) were from SIGMA-ALDRICH, and paraformaldehyde (PFA) was from ALFA AESAR*.* Recombinant human Fas-L was from R&D Systems. Mouse anti-Fas monoclonal antibody was from MERCK-MILLIPORE (Clone CH11, catalog number 05-201), Anti-HIF-1α mouse monoclonal antibody (clone H1alpha67, catalog number ab1) was from ABCAM*,* anti-CLR cytoplasmic domain goat polyclonal antibody (sc-389939) and anti-RAMP2 mouse monoclonal antibody (clone B-5, catalog number sc-365240) were from SANTA CRUZ BIOTECHNOLOGY*,* secondary PE-conjugated goat anti-mouse antibody was from CLINISCIENCES (catalog number 1036-09), and biotinylated goat anti-mouse (catalog number ab6788) and donkey anti-goat antibodies (catalog number ab6884) were from ABCAM. Ac-DEVD-AFC, Ac-IETD-AFC and Ac-LEHD-AFC for caspase-3, -8 and -9 activity measurement, respectively, and DTT were from ENZO LIFE SCIENCES. Tris(hydroxymethyl)aminomethane and NaCl were from PANCREAC. NP40 was from FLUKA. Fluorescence mounting medium was from DAKO.

### BAC culture

BACs were isolated from the carpal-metacarpal joint of daily slaughtered cows at the SOVIAM slaughterhouse (Meaux, France). Briefly, small pieces of cartilage were harvested from joints, rinsed 3 times before being minced. Small pieces (< 2 mm) were rinsed again before enzymatic digestion overnight in bacterial collagenase II (2% in DMEM *w/v*) at 37 °C^36^. Cells were passed through a nylon cell strainer (100 µm porosity) to avoid collection of non-digested cartilage before being rinsed, counted and seeded at high density (10^7^ cells in 14-cm diameter TPP Petri dishes) in 15 mL DMEM supplemented with 10% (*v/v*) FCS, 2 mM l-glutamine, 1% antibiotics and fungizone (*v/v*). Cells were cultured in a dedicated incubator (Binder CB150) at 37 °C in humidified atmosphere containing 5% CO_2_ and 3% O_2_ (hypoxic condition) or atmospheric O_2_ content (normoxic basal condition). Medium was changed twice a week for 2 weeks to obtain confluent cell culture. Cells were then starved overnight, detached by using trypsin/EDTA, rinsed twice, then counted and plated (3.10^5^ cells/well in 24-well culture plates) in starvation medium before treatment. Cells were cultured directly on plastic except for TUNEL and immunostaining experiments, for which they were cultured on glass coverslips. For TUNEL and caspase activity measurements, cells were treated for 24 h with 20 ng mL^−1^ Fas-L. In all cases, cells were pre-treated for 30 min with AM or AM(22-52) at 10^–7^ M, unless otherwise stated.

### cAMP measurement

BACs in 24-well culture plates were incubated with IBMX for 30 min with or without AM(22-52) before being stimulated with AM for another 30 min. A direct cAMP ELISA kit (ENZO) was used to determine cAMP level in BAC lysates following the manufacturer’s protocol.

### AM measurement

The AM (human) EIA Kit (PHOENIX PHARMACEUTICALS) was used to determine AM level in BAC cell culture supernatants following the manufacturer’s protocol.

### Real time quantitative PCR

Total RNA was extracted from 3.10^5^ BACs by using the MasterPure RNA Purification Kit (EPICENTRE) in accordance with the manufacturer’s instructions. RNA purity was assessed by measuring the absorbance at 260 nm/280 nm. All measured ratios were > 1.9. In total, 500 ng total RNA was reverse transcribed into cDNA by using a High Capacity cDNA Reverse Transcription kit (APPLIED BIOSYSTEMS) following the manufacturer’s instructions. After reverse transcription, the cDNA product was amplified by real-time PCR. The mRNA levels of Fas and GAPDH were determined with the double-strand specific Power SYBR Green dye system (APPLIED BIOSYSTEMS). All reactions followed the profile, 50 °C for 2 min, denaturation at 95 °C for 10 min, The second step was 40 cycles of denaturation at 95 °C for 15 s, and annealing and extension at 60 °C for 1 min (data collection was performed at the end of each annealing/extension step) (Roche Lightcycler 480 thermal cycler). The third step was a dissociation process to ensure the specificity of the amplicons by measuring their melting temperature (Tm). Primer sequences for FAS and GAPDH were determined with the Universal ProbeLibrary Assay Design Center (ROCHE APPLIED SCIENCE). Fas forward primer: 5′-AGAAGGCCTGTATCGTGAGC-3′ and reverse primer: 5′-TTTGCAATCACCGTTTTTCC-3′; GAPDH forward primer: 5′-AATTCTGGCAAAGTGGACATC-3′; and reverse primer: 5′-GACCATGTGAAGGTCAATGAA-3′. Primer efficiency was measured (2.01 and 1.95 for Fas and GAPDH, respectively). Data analysis was performed with the Lightcycler 480 software (ROCHE APPLIED SCIENCE).

### In vitro apoptosis

BAC early apoptosis was first determined by caspase-3, -8, and -9 activity measurements by using specific protease substrates as described^19^. Briefly, cells were lysed in 200 µL lysis buffer [tris(hydroxymethyl)aminomethane 10 mM, NaCl 200 mM, EDTA 5 mM, glycerol 10% (*v/v*) and NP40 1% (*v/v*) at pH 7.4] for at least 30 min at 4 °C after 24 h of Fas-L treatment with or without AM or AM(22-52). Lysates were then frozen and stored at − 20 °C. After thawing, they were centrifuged at 10,000 rpm for 10 min at 4 °C, and supernatants were collected. Caspase activity was determined by specific fluorogenic substrate cleavage (DEVD-AFC, LEHD-AFC, IETD-AFC, for caspase-3, -8 and -9, respectively). Substrates were conjugated with a fluorophore (7-amino-4-trifluoromethyl coumarin) and fluorescence was measured after its release at 505-nm emission wavelength (excitation at 400 nm) on a GloMax-Multi fluorimeter (PROMEGA). The assay was performed with 50 µL samples in duplicate incubated for 2 h at 37 °C with 50 µL reaction buffer mixed in reaction buffer [DTT 10 mM, PMSF 0.1 mM, Hepes 10 mM at pH 7.4] containing 5 µL specific substrate (1 mM). Results are expressed as arbitrary units and normalized to total protein content (BCA Protein Assay kit, PIERCE).

TUNEL assay was performed to evaluate late apoptosis. BACs cultured on glass coverslips were stained for TUNEL assay by using a commercial kit (ApopTag Red In Situ Apoptosis Detection Kit, MILLIPORE), according to the manufacturer’s protocol. Briefly, chondrocytes were fixed first with 1% paraformaldehyde (*w/v*), then with an acetic acid/ethanol solution. Cells were incubated with terminal deoxynucleotidyl transferase enzyme for 1 h at 37 °C in a humidified dark chamber and with rhodamine-coupled digoxigenin at room temperature. Finally, cells were rinsed and mounted with the appropriate medium. Image acquisition was performed on an inverted Zeiss AxioImager Z1 microscope with a × 20 objective. Cell counts were performed on four randomly chosen fields containing at least 100 cells. Results are expressed as percentage of TUNEL-positive cells versus total cell number.

### Immunostaining

Chondrocytes were fixed in 4% paraformaldehyde in DPBS concomitantly with permeabilization agent (0.1% triton X-100 in DPBS) at room temperature for 5 min, then exposed to 3% BSA for 1 h. For Fas or HIF-1α staining, cells were incubated with the antibody for 1 h (5 µg mL^−1^ and 4 µg mL^−1^ in BSA 3% respectively). After 3 washes with DPBS, goat anti-mouse biotinylated antibody (40 µg mL^−1^ in BSA 1%) was added for 30 min. Cells were incubated with streptavidin-Alexa Fluor 488 (1 µg mL^−1^ in BSA 1%) for 30 min. For CLR/RAMP2 double staining, cells were first incubated overnight with anti-CLR antibody at 4 °C (0.5 µg mL^−1^ in BSA 1%). After 3 washes with DPBS, a blocking step in 3% donkey serum was performed before donkey anti-goat biotinylated antibody (40 µg mL^−1^ in BSA 1%) was added for 30 min. Cells were then incubated with streptavidin-Alexa Fluor 488 (1 µg mL^−1^ in BSA 1%) for 30 min, then blocked with an exposure to 3% BSA for 30 min before incubation with anti-RAMP2 antibody (4 µg mL^−1^ in BSA 3%). Cells were washed 3 times with DPBS and incubated for 30 min with anti-mouse PE-conjugated goat antibody (10 µg mL^−1^ in BSA 1%). In all cases, cells were washed 3 times with DPBS before mounting and nuclei were counterstained with DAPI (1 µg mL^−1^ in H_2_O) for 5 min. Cells were observed under an inverted Zeiss AxioImager Z1 AxioCam MR3 microscope with an oil immersion × 63 objective. Staining quantifications involved use of ImageJ.

### Statistical analysis

All experiments were performed in duplicate with BACs derived at least from 6 independent animals for each experiment. The significance of results was assessed first with a non-parametric Kruskal–Wallis test followed by a post-hoc exact non-parametric and stratified Wilcoxon–Mann–Whitney test as appropriate (StatXact 7.0, Cytel Inc.). We used non-parametric statistics because of lack of normal distribution of the assessed variables (due to small number of samples), and the stratification allowed for taking into account the impact of individual variability. Difference between conditions was considered significant at *p* < 0.05.
